# Prevalence and Treatments of Movement Disorders in Prion Diseases: A Longitudinal Cohort Study

**DOI:** 10.1002/mds.29152

**Published:** 2022-07-16

**Authors:** Danielle Sequeira, Akin Nihat, Tzehow Mok, Thomas Coysh, Peter Rudge, John Collinge, Simon Mead

**Affiliations:** ^1^ National Prion Clinic University College London Hospitals NHS Foundation Trust London UK; ^2^ MRC Prion Unit at UCL Institute of Prion Diseases London UK

**Keywords:** movement disorders, prion, Creutzfeldt‐Jakob, sCJD

## Abstract

**Background:**

Prion diseases cause a range of movement disorders involving the cortical, extrapyramidal, and cerebellar systems, and yet there are no large systematic studies of their prevalence, features, associations, and responses to commonly used treatments.

**Objectives:**

We sought to describe the natural history and pharmacological management of movement disorders in prion diseases.

**Methods:**

We studied the serial examination findings, investigation results, and symptomatic treatment recorded for 700 patients with prion diseases and 51 mimics who had been enrolled onto the prospective longitudinal National Prion Monitoring Cohort study between 2008 and 2020. We performed an analysis to identify whether there were patterns of movement disorders associated with disease aetiology, *PRNP* codon 129 polymorphism, disease severity rating scales, magnetic resonance imaging (MRI) and cerebrospinal fluid (CSF) findings.

**Results:**

Gait disturbances, myoclonus, and increased tone are the most frequently observed movement disorders in patients with prion diseases. The typical pattern of early motor dysfunction involves gait disturbance, limb ataxia, impaired smooth pursuit, myoclonus, tremor, and increased limb tone. Disturbances of gait, increased tone, and myoclonus become more prevalent and severe as the disease progresses. Chorea, alien limb phenomenon, and nystagmus were the least frequently observed movement disorders, with these symptoms showing spontaneous resolution in approximately half of symptomatic patients. Disease severity and *PRNP* codon 129 polymorphism were associated with different movement disorder phenotypes. Antiepileptics and benzodiazepines were found to be effective in treating myoclonus.

**Conclusions:**

We describe the prevalence, severity, evolution, treatment, and associated features of movement disorders in prion diseases based on a prospective cohort study. © 2022 The Authors. *Movement Disorders* published by Wiley Periodicals LLC on behalf of International Parkinson and Movement Disorder Society

## Graphical abstract

We studied the serial movement disorder findings for 751 prion disease patients/mimics. We observed increasing gait disturbance, limb ataxia, impaired smooth pursuit, myoclonus, tremor, and increased limb tone to mid‐stages of disease. *PRNP* codon 129 polymorphism was a modifier. Antiepileptics and benzodiazepines were effective in treating myoclonus.

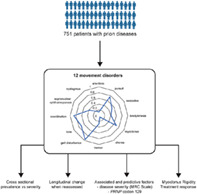

## Introduction

Human prion diseases are rare fatal neurodegenerative diseases with sporadic, genetic, and acquired etiologies, and a unifying underlying mechanism involving the misfolding of cellular prion protein (PrP^C^) into multimeric assemblies. Prions, the infectious agent of prion diseases, are thought to comprise solely or predominantly of abnormal prion protein (PrP^Sc^). Prion diseases of humans and animals show marked phenotypic heterogeneity including distinctive clinical and histopathological features.^1^, ^2^ These phenomena may in part be explained by prion strains, whereby distinct clinicopathological features are maintained when the disease is transmitted between animals or humans. The extent to which this variation in patients is determined by demographic factors, prion strains, environmental, genetic, or stochastic factors remains unclear.

Movement disorder symptoms are commonly observed in patients with prion diseases and feature in the epidemiological diagnostic criteria, and yet there are no prospective studies that describe their systematic evaluation and progression.^3^, ^4^ A wide spectrum of hyperkinetic to akinetic movement disturbances, including ataxia, myoclonus, dystonia, tremor, choreoathetosis, hemiballismus, and atypical parkinsonian syndromes, have been reported in case reports and case series.^5^, ^6^, ^7^, ^8^, ^9^ Differences in reported frequencies of these symptoms have been attributed to variations in the definition of timing of symptom onset, symptom categorization, codon 129 polymorphism population frequency, and PrP^Sc^ Western blot typing. Cerebellar ataxia and myoclonus are thought to be the most frequently reported movement disorders in prion diseases, followed by rigidity.^5^, ^6^ There is little published evidence to support prescribing for movement disorders in this context; there are no randomized controlled trials of treatments for myoclonus in prion disease.

This article aims to provide a comprehensive review of the spectrum of movement disorder symptomatology in prion disease using systematically collected, prospective, observational data from the ongoing National Prion Monitoring Cohort study, in order to provide a resource describing the variety, severity, and natural history of movement disorders in prion diseases and their mimics. We analyze predictor variables and provide evidence to justify choices of therapeutic for myoclonus, the most common treatable movement disorder in prion diseases.

## Methods

UK neurologists are requested to report suspected cases of prion disease to the National Creutzfeldt‐Jakob Disease Research & Surveillance Unit (NCJDRSU) and National Prion Clinic (NPC). All patients consecutively referred to the NPC between October 2008 and November 2020, who had been enrolled onto the National Prion Monitoring Cohort (NPMC) study, a longitudinal prospective study, were included in this analysis. Ethics approval was granted by the Scotland A Research Ethics Committee and informed consent was obtained from the patient or next of kin, according to the Mental Capacity Act 2005, as appropriate. Patients with a diagnosis of sporadic CJD (sCJD), symptomatic inherited prion disease (IPD), variant CJD (vCJD), iatrogenic CJD (iCJD), according to contemporary diagnostic criteria,^3^, ^4^ and cases that were suspected to have CJD but subsequently clinically or neuropathologically diagnosed with a non‐prion disorder, were included in this analysis. For detailed characteristics of mimics in our Cohort see Mead and Rudge (2017) and Rudge et al (2018).^10^, ^11^

Patients underwent standardized neurocognitive examinations by one member of the NPC team at initial assessment and in rapidly progressive (Stratum 1) patients at 4–8 weekly follow‐up intervals,^12^ and at 6–12 months (Stratum 2) in slowly progressive IPD patients. A report of symptoms and signs were reviewed by a panel of one to three consultant neurologists (PR, SM, JC) within 1 week. Patient data, prescribed medications, examination findings, and investigation results were recorded at each visit and coded on a database. Disease duration was estimated from first reported symptom, and stage of disease was recorded using the Medical Research Council Prion Disease Rating Scale (MRC Scale), a unidimensional scale used to objectively measure functional decline in patients with sCJD.^12^ PrP^Sc^ Western blot type, using the London classification of the electrophoretic pattern of PrP following proteinase K digestion, was also recorded.^13^ Data regarding the occurrence (binary data) and severity (numeric ordinal data) of a range of movement disorders including visual pursuit and saccades, nystagmus, limb ataxia, gait disturbance, bradykinesia, increased tone, tremor, chorea, alien limb, supranuclear ophthalmoparesis (direction of gaze palsy not recorded), and myoclonus, were collected. Bradykinesia was only coded if there was clear evidence of decrement on repetition of oppositional movement of the thumb upon the first finger, usually over five or more cycles. Cerebellar action tremor was assessed on finger‐nose and heel–shin testing and was of low frequency. It was coded abnormal if there was a failure to achieve and maintain the target on three consecutive cycles with either upper or lower limbs; on occasion it was difficult to code the relative contributions of ataxia and apraxia. In prion diseases, several phenomena are thought to contribute to disturbances in gait including increases in muscle tone, tremor, ataxia, and apraxia (defined as gait initiation impairment and loss of normal cadence with short shuffling steps).^14^ Consequently, we have included patients with any disturbance of gait in this category. Dystonic posture or movement, a rare feature in CJD, was not recorded. Furthermore, cogwheel rigidity could be caused by rigidity with any superimposed repetitive hyperkinetic disorder (parkinsonism, dystonic tremor, myoclonus) and this sign was not recorded. Missing examination data were recorded as unassessable, with data imputation not performed due to the multitude of factors possibly contributing to data not being collected, including the difficulties posed when examining patients with global dementia and other neurological impairments, patient fatigue, behavioral symptoms, limited assessment time, and erroneous omission.

Cross‐sectional movement disorder data from the first assessment following enrollment, or symptom manifestation in patients with IPD (with mutations expected to cause a sCJD‐like phenotype) who were recruited when healthy, were studied to determine the prevalence of movement disorders in patients with a rapidly progressive phenotype (Stratum 1, meaning a physician‐predicted total clinical duration of <2 years, see results for IPD mutations included in this series). We also studied the prevalence of movement disorders by MRC Scale score, to reflect the natural history and timing of the appearances of movement disorders in prion diseases. A longitudinal analysis of the data was performed using data collected at first follow‐up assessment.

Prescription data were analyzed for medications which had been prescribed to at least five patients for each movement disorder symptom. If more than one drug was prescribed for that symptom, the clinical notes were reviewed to differentiate relative clinical benefit and adverse effects of each medication. If it was not possible to distinguish relative effects, the benefit or adverse effect was attributed to both.

### Statistical Analysis

Proportion confidence intervals for prevalence were calculated using the Exact (Clopper–Pearson) method. Continuous parameters were compared using Kruskal–Wallis test. Categorical parameters were compared using *X* ^2^ test. A *P* value <0.05 was considered suggestive of significance, and *P*<0.001 was considered statistically significant in consideration of multiple testing in the predictor analysis. Multinomial and ordinal logistic regression analyses were performed to calculate the relative risk ratio of each movement disorder by disease type (including CJD mimics), codon 129 polymorphism, and MRC Scale score, controlling for differences in gender and age at onset, and the predictive effects of investigation results. The reference group was unaffected, male, with a diagnosis of sCJD, MRC 18–20, and MM at codon 129. All statistical analyses were performed using Stata SE.

## Results

A total of 870 individuals were enrolled to the NPMC between October 17, 2008 and November 8, 2020, of whom 751 patients were included in this study (48% male, 52% female, Table 1). Of the 119 patients excluded, 42 were controls, 34 were asymptomatic with IPD mutations, 29 were healthy at‐risk of IPD, and 14 were healthy at‐risk of iatrogenic CJD. The most frequent patient group were patients with sCJD (n = 548), followed by IPD (n = 123), iCJD (n = 19), and vCJD (n = 10). Fifty‐one patients who were referred with a suspected diagnosis of sCJD were subsequently given an alternative diagnosis (CJD mimic). Some 322 patients underwent a post‐mortem, which confirmed the clinical diagnoses in all cases. Nine cases were found to have coexisting dementing pathologies including Huntington's disease, Alzheimer's disease, and Lewy Body disease. The median age of disease onset was 66 years in sCJD and mimics, and younger in other prionopathies, being the lowest for vCJD (26 years). Patients with E200K IPD and sCJD had the shortest median disease durations (4 and 5 months respectively), whereas the 6‐OPRI IPD patients had the longest course of illness (median, 147 months).

**TABLE 1:** Demographics of patient groups

Parameter	Prion disease (700)	sCJD 548 (78%)	IPD					iCJD 19 (3%)	vCJD 10 (1%)	Mimics (51)	*P*
			P120L 29 (4%)	E200K 23 (3%)	6‐OPRI 21 (3%)	D178N 10 (1%)	Other IPDs 40 (6%)				
Male/female (%)	49/51	48/52	31/69	30/70	52/48	80/20	45/55	89/11	70/30	37/63	0.001
Age at onset (year), mean (median)	61.9 (64)	65.8 (66)	50.8 (53)	63.1 (64)	32.9 (35)	51.9 (53)	49.8 (46)	44.8 (45)	30.4 (26)	66.7 (66)	<0.001
Total disease duration (m), mean (median)	16	8.6 (5)	61.5 (55)	4.5 (4)	146.4 (147)	20.6 (22)	71.8 (33)	12.3 (13)	25.9 (14)	29.2 (19)	<0.001
MRC Scale score, mean (median)	8.3 (8)	7.1 (6)	15.4 (18)	8.6 (6)	12.4 (17)	17.4 (19)	11.8 (15)	12.8 (15)	12.3 (12)	8.2 (9)	<0.001
MM/MV/VV (%)	49/31/19	46/30/24	64/3 6/0	87/13/0	67/33/0	40/60/0	43/40/18	33/66/0	80/20/0	37/47/16	<0.001

*P* values are from a comparison of the values in each row between the different prion disease etiologies (ie, sCJD, IPD, iCJD, vCJD, and mimics) using Kruskal–Wallis test and *X* ^2^ test.

Abbreviations: iCJD, iatrogenic Creutzfeldt‐Jakob disease; IPD, inherited prion disease; MRC Scale, Medical Research Council Prion Disease Rating Scale; sCJD, sporadic CJD; vCJD, variant CJD.

### Natural History of Movement Disorders in Patients with a Rapidly Progressive Prion Disease Phenotype

#### Cross‐Sectional Data

**FIG 1:**
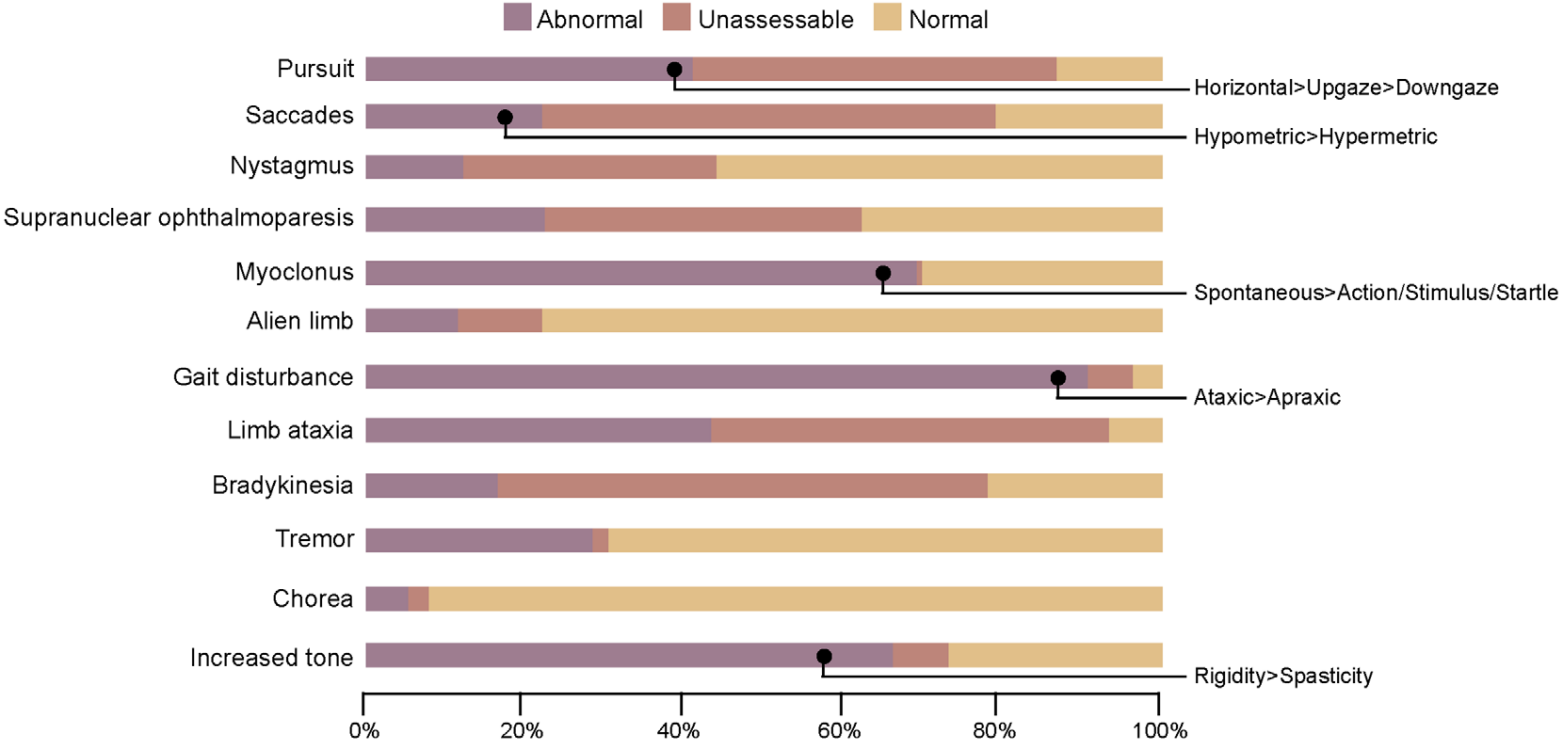
Cross‐sectional prevalence of movement disorders in prion disease. Bar chart of the prevalence of coded movement disorders in Creutzfeldt‐Jakob disease (CJD) and other rapidly progressive prion diseases at the first assessment. Supranuclear ophthalmoparesis was observed in the vertical plane. [Color figure can be viewed at wileyonlinelibrary.com]

Initially we looked at the prevalence of movement disorders at the first assessment (Fig. 1). Those expected to have rapidly progressive phenotypes included a total of 588 patients (548 sCJD and 40 with IPD: 23 E200K, 6 4‐OPRI, 5 D178N, 2 V210I, 2 E196K, 1 5‐OPRI, and 1 E211Q). At the first assessment, gait was most commonly affected, with 91% of cases manifesting an abnormal gait or being unable to mobilize independently (6% of cases were unassessable). Some 45% of patients were recorded as having an ataxic gait, compared to 32% of patients who were recorded as having an apraxic gait. Neurologists found it difficult to dichotomize the gait as either ataxic or apraxic in 23% of those patients who were able to mobilize (independently or with assistance). Sixty‐nine percent of patients exhibited myoclonus, which occurred spontaneously in a greater proportion of patients (51%) compared to stimulus sensitive (45%) and startle (43%) myoclonus. Sixty‐six percent of patients were found to have increased limb tone. Increased tone in the upper limbs was categorized as rigidity in 73% of patients, and as spasticity in 17% of patients, with 10% of patients having been recorded to have both spasticity and rigidity contributing to their hypertonia. Of the patients with increased tone of their lower limbs (fewer cf upper limb), 56% were categorized as having rigidity, 35% spasticity, and 9% both rigidity and spasticity. Eye movements, coordination, and bradykinesia were unassessable in a third to half of patients. Pursuit was more affected in the horizontal plane and on upgaze compared to down gaze. Saccades were predominantly hypometric and were recorded in 90% of affected patients compared to the 7% of patients who were recorded as having hypermetric saccades, and 2% were recorded as having both. To what extent the saccadic abnormality was an oculo‐motor apraxia was not determined and head movement compensation for delayed saccade generation was not recorded. Chorea was the least prevalent movement disorder, recorded in only 5% of cases.

**FIG 2:**
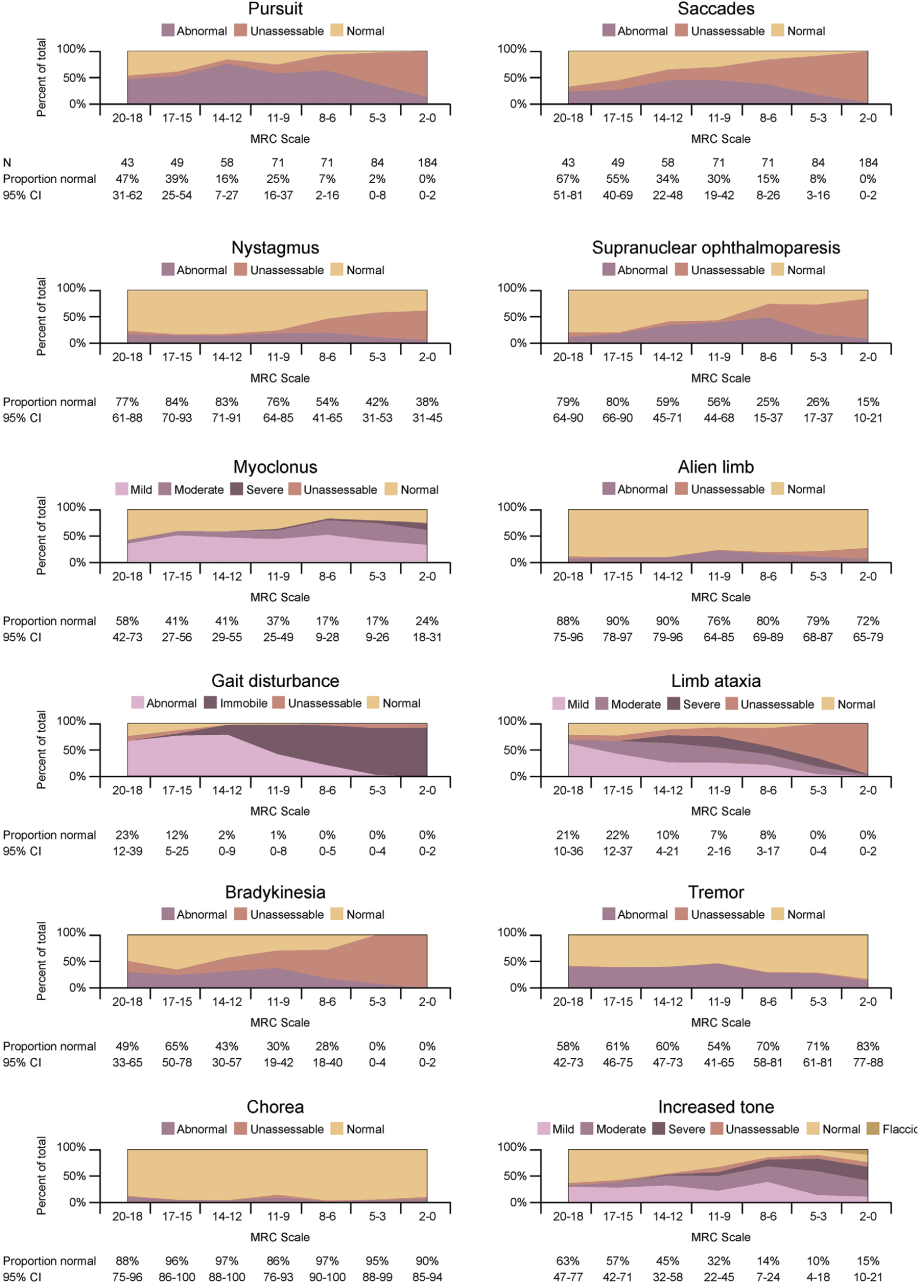
Natural history of movement disorders in prion disease. These charts show the cross‐sectional prevalence of movement disorders in rapidly progressive prion diseases at first assessment, by disease severity (measured by Medical Research Council Prion Disease Rating Scale [MRC Scale] 20–0). [Color figure can be viewed at wileyonlinelibrary.com]

We went on to look at the correlation between overall disease severity measured by the MRC Scale at first assessment with the prevalence of movement disorders (Fig. 2). Limb ataxia and gait disturbance were the most prevalent movement disorders present at the very earliest stage of disease (MRC Scale 18–20 meaning minimal impairment of activities of daily living, 70% and 67% of cases, respectively), followed by impaired smooth pursuit (47%). As a generality, the prevalences of all of the movement disorders appeared to increase until the mid‐stages of the disease. In the latter half of the disease course, signs which can only be elicited through patient cooperation become increasingly unassessable, while the remaining movement disorders, excluding tremor, continue to increase in prevalence and severity.

Myoclonus reached a maximal prevalence at an MRC Scale score between 8 and 3 (83% of cases, 95% CI, 76–89), at which stage most patients are unable to mobilize independently, are doubly incontinent, fully dependent for activities of daily living, and have severely impaired cognition.^12^ At an MRC Scale score between 5 and 3, 43% (95% CI, 32–54) of patients manifest moderate‐to‐severe myoclonus and 69% (95% CI, 58–79) manifest moderate‐to‐severe hypertonia. At the most advanced stage of the disease, MRC 2–0, the overall prevalence of myoclonus and increased tone decreased, although this group had the highest proportion of patients recorded as having severe signs. Ten percent of cases were found to have hypotonia.

### Longitudinal Data

Correlative cross‐sectional data can be misleading because patients with different disease types and more rapid rates of progression are expected to present with more advanced disease. We therefore studied 220 patients who were followed up by clinical examination at least once, after a median interval of 5 weeks (4 days–15 months). The remaining 368 patients (63%) died following initial assessment. Patients had a mean MRC Scale score of 11.0 (median 12, range 0–20) at initial assessment, which was lower at follow‐up by an average MRC Scale score of 3.7. The purpose was to enquire whether longitudinal changes in movement disorders were in keeping with the cross‐sectional data.

**FIG 3:**
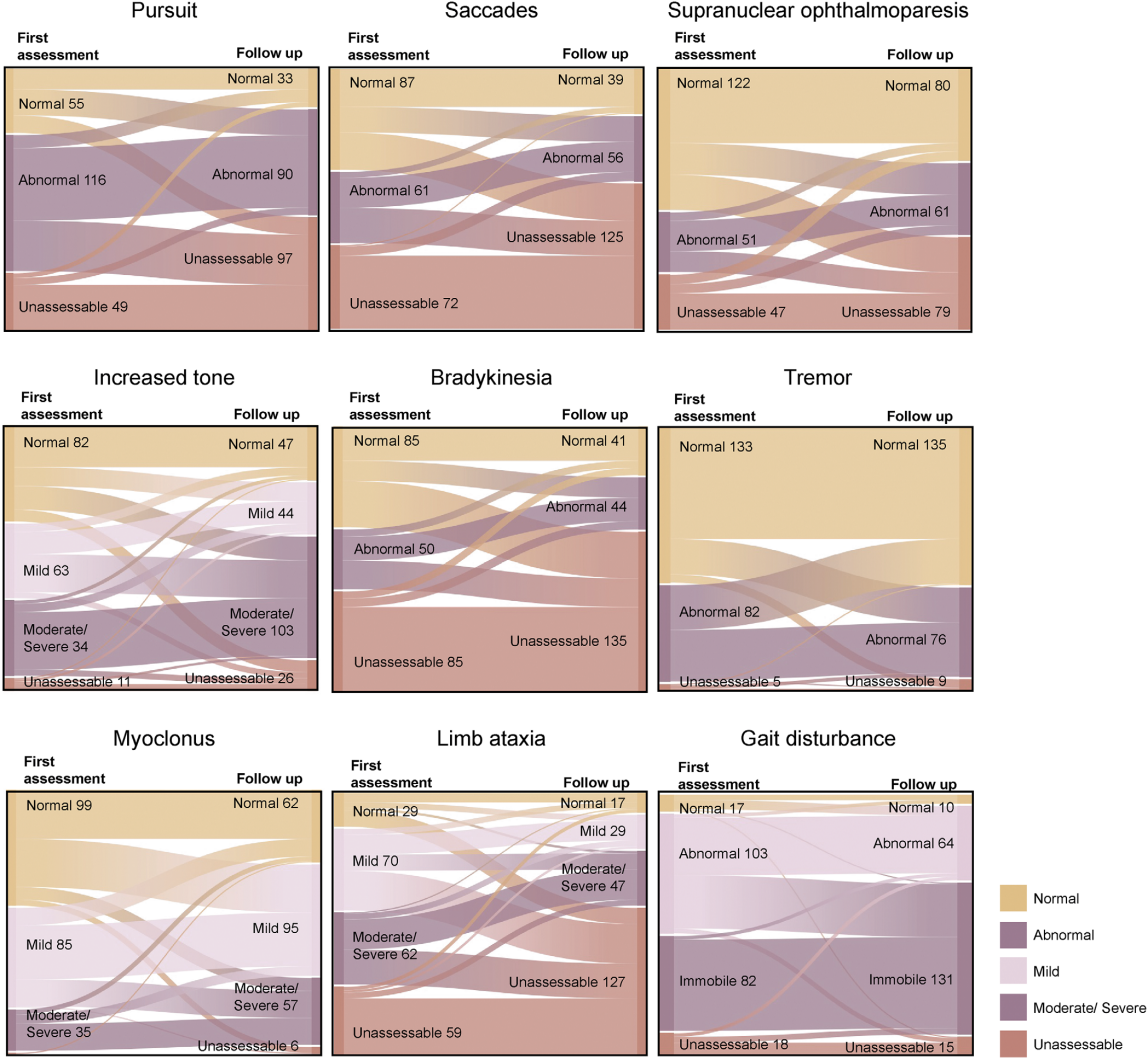
Natural history of movement disorders in prion disease. These Sankey charts show the prevalence of movement disorders in rapidly progressive prion diseases at first assessment and second assessment. [Color figure can be viewed at wileyonlinelibrary.com]

Over the course of clinical follow‐up, some movement disorders became much more prevalent (Fig. 3, Fig. S1); 40–53% of patients who had initially been unaffected by disturbances of gait, myoclonus, hypertonia, or impaired smooth pursuit subsequently developed the corresponding movement disorder at follow‐up. Nystagmus, chorea, and alien limb phenomenon remained uncommon, with only 5–9% of patients developing these de novo on follow‐up (Fig. S1). As a generality, the longitudinal analysis confirmed the findings of the cross‐sectional analysis in that as the condition progresses, most movement disorders worsen and then become difficult to assess when the condition is advanced (Fig. 3). Exceptions are the active and hyperkinetic movement disorders where 41–67% of patients recorded as having nystagmus, tremor, chorea, or alien limb at initial assessment, had spontaneous resolution of the respective movement disorder at follow‐up. Some 25–49% of patients with eye movement disorders, bradykinesia, or limb ataxia were unassessable at follow‐up. Also 45% and 51% of patients with gait disturbance or mildly increased tone, respectively, progressed to more severe disease at follow‐up, whereas this increase in severity was only observed in 27% of patients with mild myoclonus, possibly because of treatment effects (see later).

### Factors Affecting Movement Disorder Phenotype in Prion Diseases

**FIG 4:**
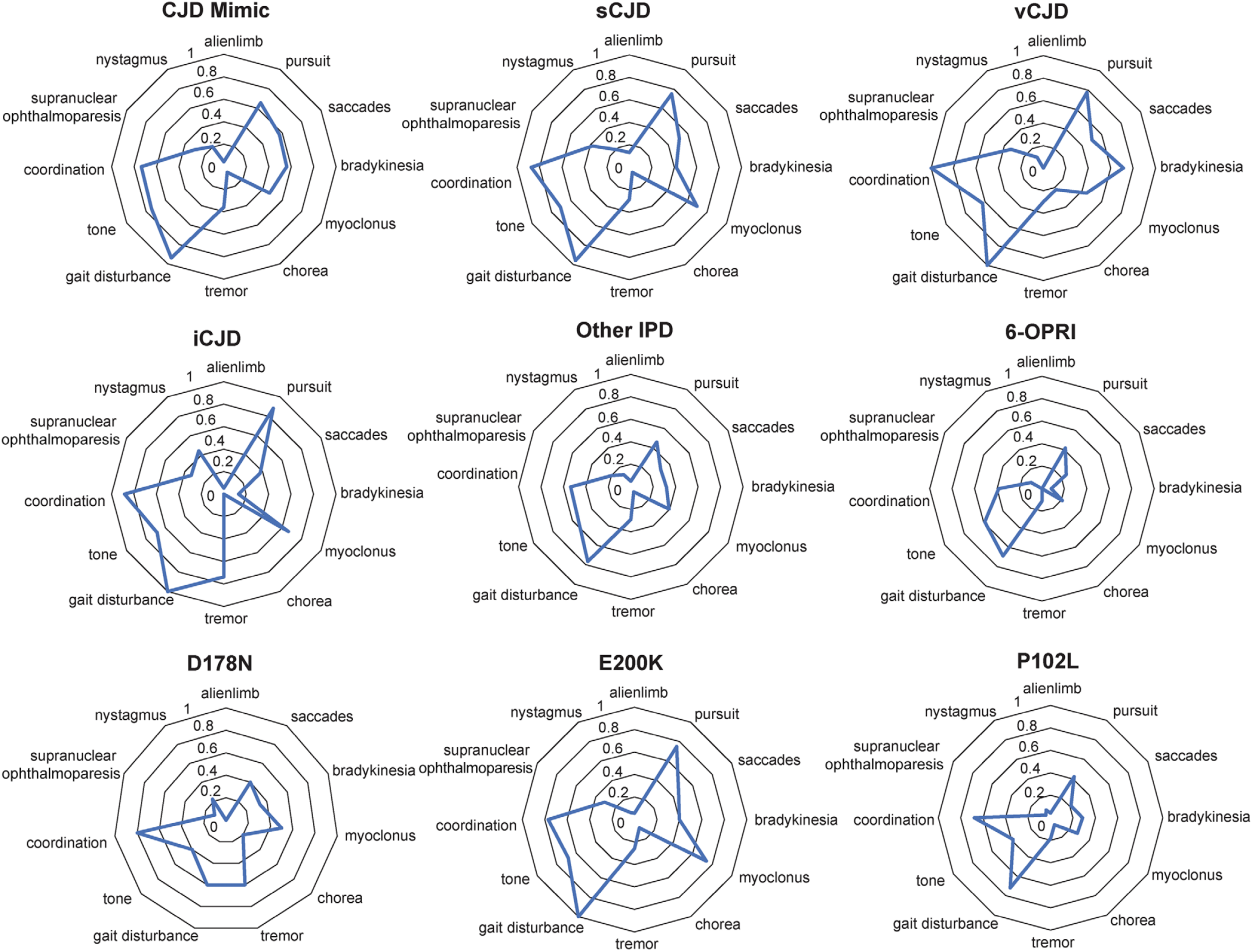
Radar plot of disease subtypes. These plots illustrate the proportion of patients in each diagnostic group who had the movement disorder recorded. These show strikingly similar patterns for sporadic Creutzfeldt‐Jakob disease (sCJD), variant CJD (vCJD), and E200K inherited prion disease (IPD), and notable differences with other IPDs and iatrogenic CJD (iCJD). [Color figure can be viewed at wileyonlinelibrary.com]

Data visualization of the cross‐sectional movement disorder profiles of the 751 studied patients (Fig. 4) suggested that patients with iCJD, 6‐OPRI, D178N, and P102L IPDs may have distinctive movement disorder profiles, but other subtypes, notably sCJD, CJD mimics, vCJD, and E200K IPD, seemed remarkably similar to each other. The visualization can be deceptive, however, because slowly progressive forms of inherited prion disease are typically diagnosed at much earlier stages than CJD. A formal analysis of each movement disorder by disease subtype was done by ordinal and categorical logistic regression with the dependent variable as each movement disorder (coded 0 = normal, 1 = abnormal, 2 = unassessable), and disease subtype, gender, age of onset, codon 129, and MRC Scale as predictors (Table S1). In both the ordinal and categorical analysis there were trends for reduced risk of myoclonus in P102L and CJD mimics (*P* = 0.04, 0.02), and increased risk of tremor in iCJD (*P* = 0.007), and no robust evidence for other movement disorders (*P* > 0.001). In the sCJD subgroup alone, we found evidence (*P* < 0.001) that myoclonus (more prevalent in MM and VV genotypes), tremor (MV and VV genotypes), supranuclear ophthalmoparesis (MV genotype), and alien limb phenomenon (MM genotype) were modified by *PRNP* codon 129 genotype (Figs. S2 and S3). As discussed earlier, unsurprisingly MRC Scale was a strong predictor for all movement disorders, whereas age and gender did not have predictor effects.

Analysis of diagnostic investigations (presence of cortical ribbon, pulvinar sign, basal ganglia, or thalamic abnormality; periodic sharp wave complexes, generalized slowing on electroencephalogram (EEG); cerebrospinal fluid (CSF)‐positive 14–3‐3 and RT‐QuIC) was unrewarding in robust movement disorder associations (Table S2). There was a non‐significant higher frequency of the pulvinar sign in patients with bradykinesia, chorea, and gait disturbance, which is in keeping with previous reports that chorea occurs more frequently in patients with vCJD.^8^, ^9^ Analysis of PrP^Sc^ strain type was limited by this being available in only 19% of sCJD cases, and showed no statistically significant associations, but is of course underpowered because PrP^Sc^ type is collinear with codon 129 genotype, meaning it would be very difficult to demonstrate independent associations (Fig. S2).

### Pharmacological Treatment of Movement Disorders in Prion Diseases

Medications had been prescribed for several movement disorder symptoms including myoclonus, increased tone, parkinsonism, tremor, and dystonia; however, there were only a few cases treated for each of the latter three symptoms. Co‐beneldopa had been trialed in one patient with parkinsonism; clonazepam and propranolol had been prescribed for tremor; and levetiracetam had been prescribed in one patient to treat dystonia.

A total of 157 patients with myoclonus had been prescribed medications (Table 2). Eighty prescriptions were written for antiepileptics, and 97 prescriptions were written for benzodiazepines. Levetiracetam and clonazepam were the most frequently prescribed medications and a clinical benefit was documented in 86% and 95% of patients, respectively. One patient appeared to develop tolerance to midazolam. Sedation was the most commonly encountered side effect across both drug classes, occurring in 23% of patients who were prescribed clonazepam. Other medications prescribed by others to treat myoclonus included carbamazepine (often said to worsen myoclonus ) (n = 4), diazepam (n = 4), lorazepam (n = 3), clobazam (n = 2), piracetam (n = 1), gabapentin (n = 1), phenytoin (n = 1), and baclofen (n = 1).

**TABLE 2:** Drug treatment of myoclonus and hypertonia

Movement disorder and medication	Prescriptions (N)	Max daily dose (mg)	Clinical benefit	Adverse effects	No follow‐up as patient died (N)	Reason for discontinuation					Predominant side effects noted
						Adverse effects	Lack of benefit	Switched to alternative	Ongoing at last assessment	Symptoms resolved	
			[N (%)]	[N (%)]		[N (%)]	[N (%)]	[N (%)]	[N (%)]	[N (%)]	
Myoclonus											
Antiepileptics											
Levetiracetam	72	3000	43 (86)	3 (6)	22	1 (1)	1 (1)	2 (3)	67 (93)	1 (1)	Altered alertness (n = 3), itch (n = 1)
Sodium valproate	8	1500	6 (86)	0	1	0	0	0	8 (100)	0	
Benzodiazepines											
Clonazepam	63	9	42 (95)	10 (23)	19	2 (3)	0	2 (3)	58 (92)	1 (2)	Drowsiness
Midazolam	34	85	17 (94)	1 (5)	16	0	0	0	34 (100)	0	Sedation
Increased tone											
Baclofen	10	40	2 (22)	1 (11)	1	1 (1)	5 (50)	0	4 (40)	0	Drowsiness

Eighteen patients were prescribed medications to treat increased tone. Baclofen was prescribed in 10 cases, showing a clinical benefit in only two cases. One case died before follow‐up. One patient suffered drowsiness as an adverse effect and the medication was discontinued. The remaining prescriptions trialled for increased tone included peripherally acting muscle relaxants such as dantrolene (n = 4); other centrally acting muscle relaxants such as tizanidine (n = 1); benzodiazepines such as midazolam (n = 1), diazepam (n = 1), and lorazepam (n = 1); and dopamine agonists, co‐beneldopa (n = 1) and co‐careldopa (n = 1).

## Discussion

We describe the movement disorder characteristics of a prospective longitudinal cohort of patients diagnosed with prion diseases and their mimics, examined by the same specialist team in the UK. We report clinical features at diagnostic and follow‐up assessments, predictor variables, and evidence for treatment benefits of two of the more frequent and problematic movement disorders encountered. These data may be useful as a natural history resource that might help understanding of atypical/emergent disease types or modification of the disease by future treatments.

Compared with previous studies^5^, ^6^, ^9^ this is the largest cohort analyzed for movement disorders in prion diseases. A prospective systematic study by a single team (with limited rotation of team members) should be less biased than a retrospective case series that records symptoms and signs documented by different physicians across multiple sites. Ours is the first study in prion disease movement disorders to report longitudinal follow‐up data, important in prion diseases because cross‐sectional data are biased by disease rapidity (ie, more rapidly progressive patients have more advanced disease at presentation). We have assessed responses to treatment in a sample reasonably powered to detect strong effects.

We also recognise some limitations. The Cohort study did not recruit all patients diagnosed with prion disease in the UK during the study period, excluding those who declined to participate in research, those moribund at presentation, and those only diagnosed post‐mortem. Recruited patients therefore represent those who are examinable and who might potentially be recruited to clinical trials. Not all patients progressed to post‐mortem confirmation. Whilst investigations in CJD now highly accurately predict diagnosis,^3^ the absence of brain tissue for many patients in the study means we had only low power for analysis of PrP^Sc^ Western blot type as a determinant of movement disorder phenotype. It would be useful to have a reliable way to determine PrP^Sc^ type in living patients. In the analysis of treatment effects, we only used treating clinician judgement as an outcome measure, and our conclusions are not as reliable as a randomized placebo‐controlled clinical trial.

These data have other potential uses beyond the scope of this article. We have written separately on the use of the neurological examination to develop a severity scale,^15^ complementary to the MRC Scale^12^; and additionally on the use of multimodal data to predict survival and increased care needs.^16^ These subjects are therefore not developed in this article.

The prevalence of movement disorders varies strikingly with disease progression. We were able to evaluate this effect with the use of the MRC Scale of overall disease progression and with longitudinal examination data. At the earliest stage of disease, eye movement, limb ataxia, and gait disturbances are most prevalent. Interrupted smooth pursuit, tremor, myoclonus, and increased tone rapidly become more frequent towards the middle stages of disease. Analysis of longitudinal data confirmed that the active or hyperkinetic movement disorders resolved spontaneously in most patients while gait disturbance and hypertonia increased. In patients unaffected by specific movement disorders, most had emergent gait disturbance, hypertonia, myoclonus, or interrupted smooth pursuit at follow‐up. Nearly two‐thirds of patients with immobility, alien limb, moderate‐to‐severe myoclonus, or hypertonia died within the 6‐weekly follow‐up period.

In rapidly progressive prion diseases, overall disease severity and codon 129 polymorphism were causal factors to a much greater extent than the prion disease aetiology. This observation can be extended to the mixed aetiologies of the CJD mimics group, which had a strikingly similar movement disorder profile to sCJD, emphasizing the relative importance of magnetic resonance imaging (MRI) and CSF investigations versus clinical features in the differential diagnosis. Comparable to other publications,^5^, ^6^, ^9^ gait disturbances and myoclonus were the most frequently encountered movement disorders in patients with rapidly progressive prion diseases. Unlike earlier studies^1^, ^6^ that described increased frequencies of ataxia and rigidity in valine homozygotes and MV heterozygotes, we found an increased risk of supranuclear ophthalmoparesis in patients with MV at codon 129, tremor in patients with MV and VV, and alien limb phenomenon in patients with MM. These differences may reflect the different patient populations, or the definitions of signs used by physicians.

Only case reports of treatment of movement disorders have previously been published. Oral prednisolone has been trialled for focal dystonia and high‐dose levodopa for parkinsonism, with no effect.^17^, ^18^ Haloperidol was described as efficacious for treatment of choreic movements and dystonic posture.^19^ Our study does not provide suggestions for the efficacious management of increased tone in patients with prion diseases. We found that rigidity more commonly affected limb tone than spasticity, particularly in the upper limbs, which may explain why antispasticity agents seemed to be ineffective. Furthermore, our record of medications prescribed for increased tone may be an under‐representation given the occurrence of severe rigidity late in the disease following which a further longitudinal assessment was unlikely to have been undertaken. Additionally, as the focus of treatment in prion diseases is on minimizing disability and delivering high‐quality end‐of‐life care, often treatment is not indicated for rigidity, as the disability caused is often minimal at the stage when the patient's rigidity is most severe, as they are akinetic and patients do not appear to be in pain. We do, however, demonstrate that levetiracetam and benzodiazepines are effective in treating myoclonus based on clinical judgement in 91% of patients. Lee et al^20^ previously described ineffective trials of valproate, topiramate, gabapentin, lamotrigine, and lorazepam in a single case report.

In conclusion, we report a systematic evaluation of movement disorders in a large cohort of prion disease patients and their mimics. These natural history data may be of some use in the differential diagnosis and management of patients, and as a reference dataset that might be used to evaluate disease subtypes and the effects of future treatments.

## Author Contributions

SM, JC, and DS conceived and designed the study. DS and SM analyzed the data. SM, DS, AN, THM, and PR were involved in patient recruitment, and all authors reviewed the analysis and final manuscript.

## Conflicts of interest

JC is a director of D‐Gen Ltd, an academic company working in the field of prion disease diagnosis, decontamination, and therapeutics.

## Financial Disclosures

DS was supported by the UCLH BRC grant. THM was supported by a Fellowship award from Alzheimer's Society, UK (grant number 341 [AS‐CTF‐16b‐007]). AN was supported by a Medical Research Council Clinical Research Training Fellowship (grant number MR/P019862/1). THM and AN are also supported by CJD Support Network UK Research Support Grants. JC and SM are NIHR Senior Investigators.

## Funding Sources

The study was funded by the Medical Research Council (UK) and National Institute for Health Research's Biomedical Research Centre at UCLH NHS Foundation Trust (project number 541735).

## Supporting information


**FIG. S1.** Natural history of movement disorders in prion disease. These Sankey charts show the prevalence of movement disorders in rapidly progressive prion diseases at first assessment and second assessment.

**FIG. S2.** Radar plot of prion protein (PrP) scrapie subtypes. These plots illustrate the proportion of patients in each molecular group who had the movement disorder recorded. These show strikingly similar patterns for PrP Scrapie types (by London classification system). Note however these are small sample sizes compared with the study group as a whole.

**FIG. S3.** Radar plot of *PRNP* codon 129 genotypes. These plots illustrate the proportion of patients in each genotype group who had the movement disorder recorded. These show distinct movement disorders for the codon 129 MV genotype, with a lower prevalence of eye movement disorders, bradykinesia, and myoclonus.

**Table S1**. Results of Logistic Regression Analysis

**Table S2** Results of ordinal logistic regression model for investigations. We fitted models that included age, Medical Research Council Prion Disease Rating Scale (MRC Scale), gender, disease type, and investigation status to predict each movement disorder (recorded as absent/present/unassessable). Each investigation was recorded as abnormal/normal/not assessed. Coefficients for the effect of the investigation are shown in each cell (in orange, *P* = 0.01–0.05; in yellow, *P* = 0.001–0.01; no colour, *P* > 0.05). Taking multiple testing into account we concluded no statistically significant associations (*P <* 0.001).

## Data Availability

The data that support the findings of this study are available on request from the corresponding author. The data are not publicly available due to privacy or ethical restrictions.
