# Supplementary figures and images for: Prevalence and Treatments of Movement Disorders in Prion Diseases: A Longitudinal Cohort Study
Source: Mov Disord. 2022 Jul 16;37(9):1893–903. doi: 10.1002/mds.29152 (PMC9543300; doi:10.1002/mds.29152)

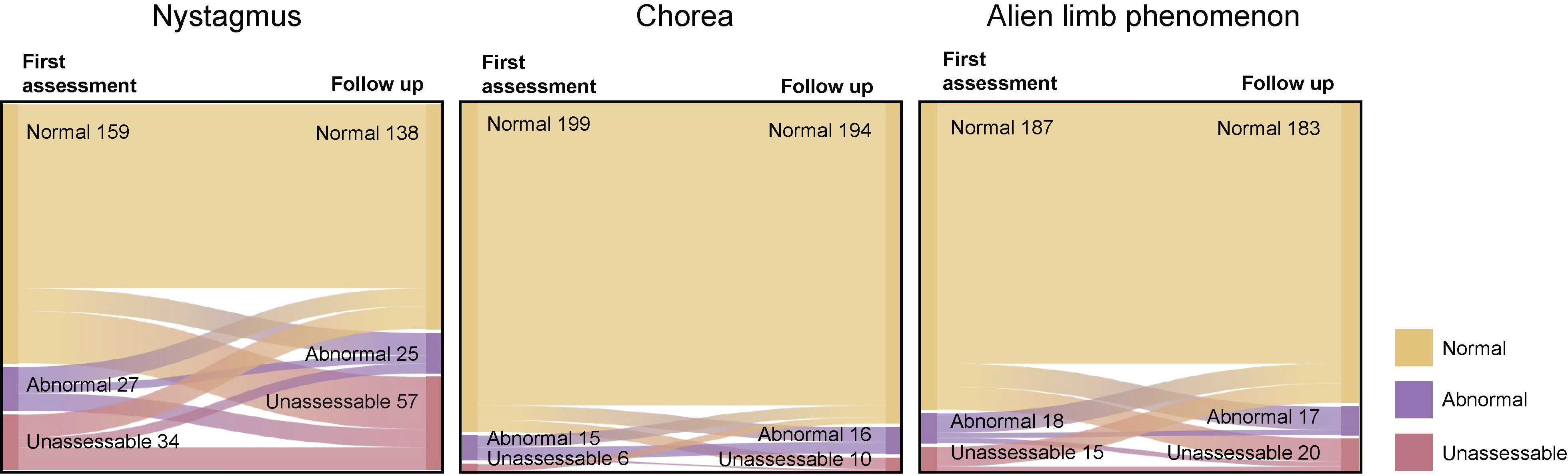

Supplement: Supplementary file 1 — FIG. S1. Natural history of movement disorders in prion disease. These Sankey charts show the prevalence of movement disorders in rapidly progressive prion diseases at first assessment and second assessment. [file MDS-37-1893-s003.tif]

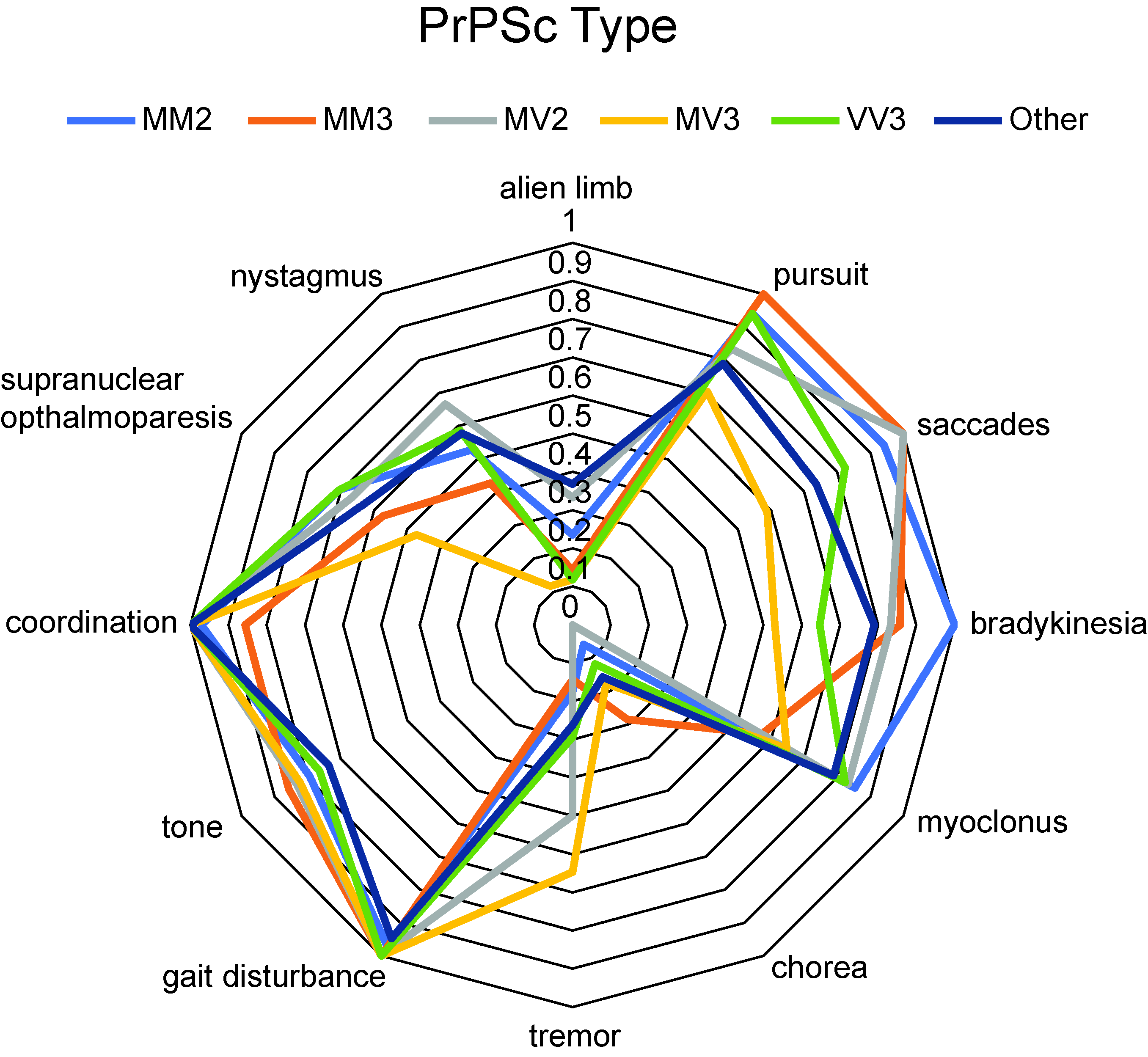

Supplement: Supplementary file 2 — FIG. S2. Radar plot of prion protein (PrP) scrapie subtypes. These plots illustrate the proportion of patients in each molecular group who had the movement disorder recorded. These show strikingly similar patterns for PrP Scrapie types (by London classification system). Note however these are small sample sizes compared with the study group as a whole. [file MDS-37-1893-s001.tif]

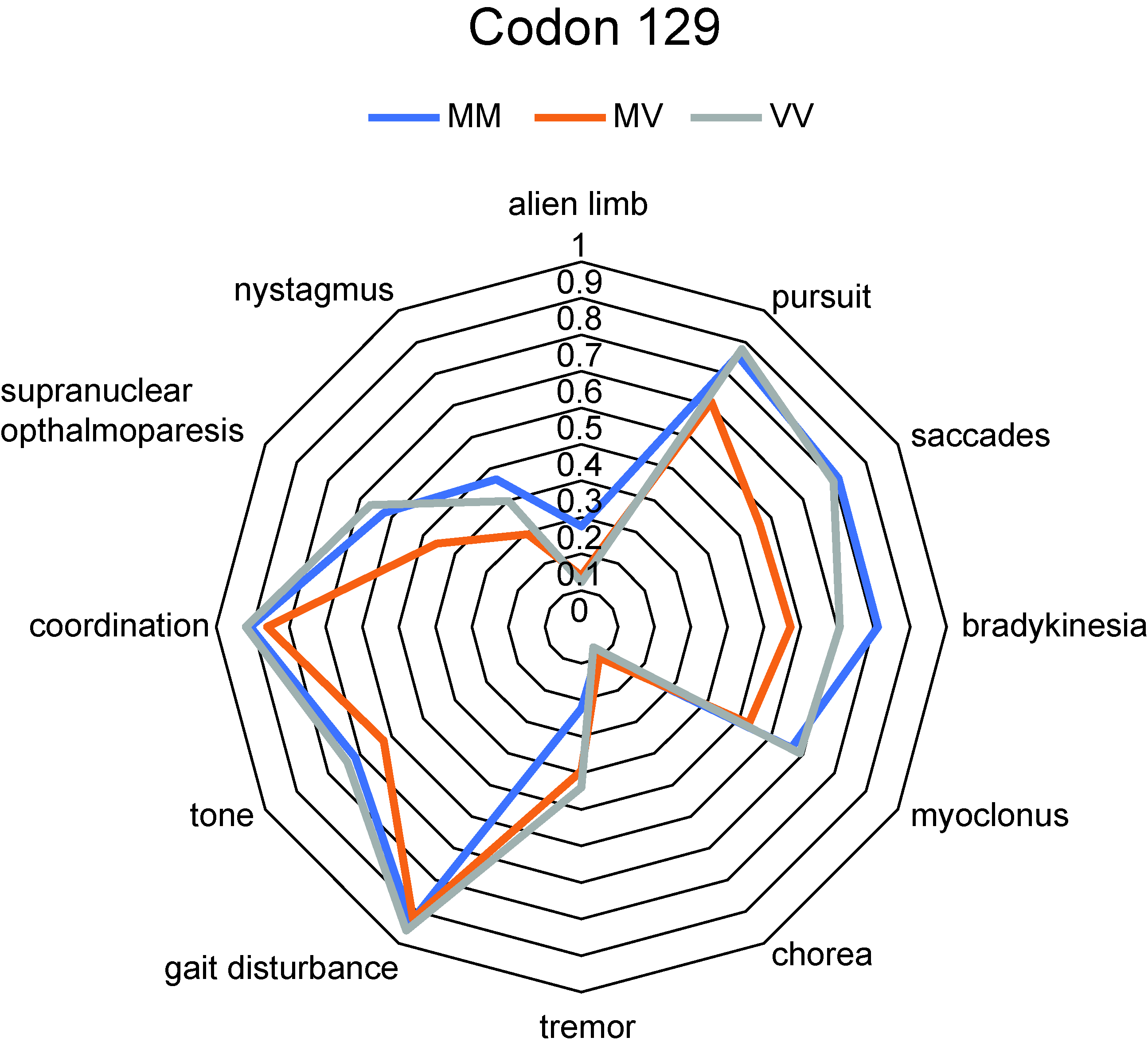

Supplement: Supplementary file 3 — FIG. S3. Radar plot of PRNP codon 129 genotypes. These plots illustrate the proportion of patients in each genotype group who had the movement disorder recorded. These show distinct movement disorders for the codon 129 MV genotype, with a lower prevalence of eye movement disorders, bradykinesia, and myoclonus. [file MDS-37-1893-s002.tif]
