# Supplementary material for: Prevalence and Treatments of Movement Disorders in Prion Diseases: A Longitudinal Cohort Study
Source: Mov Disord. 2022 Jul 16;37(9):1893–903. doi: 10.1002/mds.29152 (PMC9543300; doi:10.1002/mds.29152)
Supplement: Supplementary file 4 — Table S1. Results of Logistic Regression Analysis [file MDS-37-1893-s005.docx]

|  | **vCJD** | **iCJD** | **Other IPDs** | **6OPRI** | **D178N** | **E200K** | **P102L** | **CJD Mimic** | **Age** | **Gender** | **MRC Scale** | **MV** | **VV** |
| --- | --- | --- | --- | --- | --- | --- | --- | --- | --- | --- | --- | --- | --- |
| **Pursuit** |  |  |  |  |  |  |  |  |  |  |  |  |  |
| Coefficient | 0.10 | 0.64 | -0.09 | 0.53 | 0.99 | 0.35 | -0.53 | 1.27 | 0.01 | 0.08 | -0.25 | -0.86 | -0.57 |
| Standard Error | 0.74 | 0.54 | 0.38 | 0.58 | 0.75 | 0.51 | 0.46 | 0.70 | 0.01 | 0.18 | 0.02 | 0.21 | 0.24 |
| Z | 0.13 | 1.18 | -0.25 | 0.92 | 1.33 | 0.69 | -1.17 | 1.83 | 1.00 | 0.44 | -13.18 | -4.02 | -2.37 |
| P | 0.89 | 0.24 | 0.81 | 0.36 | 0.18 | 0.49 | 0.24 | 0.07 | 0.32 | 0.66 | 0.00 | 0.00 | 0.02 |
| Lower 95% CI Coefficient | -1.34 | -0.42 | -0.85 | -0.60 | -0.47 | -0.64 | -1.43 | -0.09 | -0.01 | -0.28 | -0.28 | -1.27 | -1.04 |
| Upper 95% CI Coefficient | 1.54 | 1.71 | 0.66 | 1.67 | 2.46 | 1.35 | 0.36 | 2.63 | 0.03 | 0.44 | -0.21 | -0.44 | -0.10 |
|  |  |  |  |  |  |  |  |  |  |  |  |  |  |
| **Saccades** |  |  |  |  |  |  |  |  |  |  |  |  |  |
| Coefficient | -0.12 | -0.14 | -0.21 | 0.43 | 0.55 | 0.01 | 0.83 | 0.46 | 0.01 | -0.20 | -0.28 | -0.87 | -0.67 |
| Standard Error | 0.74 | 0.62 | 0.41 | 0.63 | 0.74 | 0.56 | 0.48 | 0.64 | 0.01 | 0.20 | 0.02 | 0.23 | 0.25 |
| Z | -0.16 | -0.23 | -0.51 | 0.68 | 0.74 | 0.02 | 1.73 | 0.72 | 1.17 | -1.00 | -13.99 | -3.83 | -2.62 |
| P | 0.87 | 0.82 | 0.61 | 0.50 | 0.46 | 0.98 | 0.08 | 0.47 | 0.24 | 0.32 | 0.00 | 0.00 | 0.01 |
| Lower 95% CI Coefficient | -1.56 | -1.35 | -1.02 | -0.81 | -0.89 | -1.08 | -0.11 | -0.79 | -0.01 | -0.58 | -0.32 | -1.31 | -1.17 |
| Upper 95% CI Coefficient | 1.32 | 1.07 | 0.60 | 1.67 | 1.99 | 1.11 | 1.76 | 1.71 | 0.03 | 0.19 | -0.24 | -0.42 | -0.17 |
|  |  |  |  |  |  |  |  |  |  |  |  |  |  |
| **Bradykinesia** |  |  |  |  |  |  |  |  |  |  |  |  |  |
| Coefficient | 0.64 | -0.69 | 0.49 | -0.16 | 0.79 | -0.17 | 0.25 | -0.28 | 0.01 | 0.24 | -0.30 | -1.14 | -1.15 |
| Standard Error | 0.76 | 0.68 | 0.45 | 0.69 | 0.73 | 0.59 | 0.50 | 0.71 | 0.01 | 0.21 | 0.02 | 0.24 | 0.29 |
| Z | 0.85 | -1.02 | 1.09 | -0.23 | 1.08 | -0.29 | 0.51 | -0.40 | 1.06 | 1.15 | -13.68 | -4.66 | -3.98 |
| P | 0.40 | 0.31 | 0.28 | 0.82 | 0.28 | 0.77 | 0.61 | 0.69 | 0.29 | 0.25 | 0.00 | 0.00 | 0.00 |
| Lower 95% CI Coefficient | -0.84 | -2.02 | -0.40 | -1.52 | -0.65 | -1.34 | -0.72 | -1.67 | -0.01 | -0.17 | -0.35 | -1.62 | -1.72 |
| Upper 95% CI Coefficient | 2.13 | 0.64 | 1.38 | 1.20 | 2.23 | 0.99 | 1.23 | 1.11 | 0.03 | 0.66 | -0.26 | -0.66 | -0.58 |
|  |  |  |  |  |  |  |  |  |  |  |  |  |  |
| **Myoclonus** |  |  |  |  |  |  |  |  |  |  |  |  |  |
| Coefficient | 0.28 | 0.94 | -0.49 | -0.69 | 0.43 | 0.44 | -0.97 | -1.74 | 0.01 | -0.06 | -0.08 | -0.43 | 0.11 |
| Standard Error | 0.78 | 0.62 | 0.39 | 0.62 | 0.71 | 0.51 | 0.48 | 0.72 | 0.01 | 0.19 | 0.02 | 0.22 | 0.26 |
| Z | 0.36 | 1.53 | -1.25 | -1.12 | 0.61 | 0.86 | -2.03 | -2.42 | 1.33 | -0.30 | -4.86 | -1.96 | 0.43 |
| P | 0.72 | 0.13 | 0.21 | 0.26 | 0.54 | 0.39 | 0.04 | 0.02 | 0.18 | 0.77 | 0.00 | 0.05 | 0.67 |
| Lower 95% CI Coefficient | -1.24 | -0.27 | -1.26 | -1.91 | -0.95 | -0.56 | -1.90 | -3.15 | -0.01 | -0.43 | -0.11 | -0.85 | -0.39 |
| Upper 95% CI Coefficient | 1.80 | 2.15 | 0.28 | 0.52 | 1.82 | 1.44 | -0.03 | -0.33 | 0.03 | 0.31 | -0.05 | 0.00 | 0.61 |
|  |  |  |  |  |  |  |  |  |  |  |  |  |  |
| **Chorea** |  |  |  |  |  |  |  |  |  |  |  |  |  |
| Coefficient | 1.29 | -0.36 | 0.24 | 0.51 | 1.17 | 0.28 | 0.32 | 0.37 | -0.02 | 0.11 | -0.02 | 0.33 | 0.06 |
| Standard Error | 0.92 | 1.11 | 0.62 | 0.83 | 0.87 | 0.78 | 0.72 | 1.07 | 0.02 | 0.31 | 0.03 | 0.35 | 0.45 |
| Z | 1.41 | -0.33 | 0.38 | 0.62 | 1.35 | 0.36 | 0.44 | 0.34 | -1.03 | 0.36 | -0.93 | 0.93 | 0.13 |
| P | 0.16 | 0.74 | 0.70 | 0.54 | 0.18 | 0.72 | 0.66 | 0.73 | 0.30 | 0.72 | 0.36 | 0.35 | 0.90 |
| Lower 95% CI Coefficient | -0.51 | -2.53 | -0.99 | -1.12 | -0.53 | -1.25 | -1.09 | -1.73 | -0.05 | -0.50 | -0.07 | -0.37 | -0.82 |
| Upper 95% CI Coefficient | 3.08 | 1.80 | 1.46 | 2.15 | 2.88 | 1.81 | 1.72 | 2.46 | 0.01 | 0.73 | 0.03 | 1.02 | 0.93 |
|  |  |  |  |  |  |  |  |  |  |  |  |  |  |
| **Tremor** |  |  |  |  |  |  |  |  |  |  |  |  |  |
| Coefficient | 0.04 | 1.43 | 0.09 | -0.09 | 0.70 | 0.23 | -0.18 | 0.41 | 0.00 | -0.21 | 0.03 | 0.54 | 1.00 |
| Standard Error | 0.79 | 0.53 | 0.40 | 0.63 | 0.67 | 0.51 | 0.49 | 0.65 | 0.01 | 0.19 | 0.02 | 0.22 | 0.25 |
| Z | 0.05 | 2.70 | 0.22 | -0.15 | 1.04 | 0.45 | -0.36 | 0.64 | -0.15 | -1.12 | 2.07 | 2.40 | 4.06 |
| P | 0.96 | 0.01 | 0.82 | 0.88 | 0.30 | 0.65 | 0.72 | 0.53 | 0.88 | 0.26 | 0.04 | 0.02 | 0.00 |
| Lower 95% CI Coefficient | -1.50 | 0.39 | -0.69 | -1.33 | -0.62 | -0.77 | -1.14 | -0.86 | -0.02 | -0.59 | 0.00 | 0.10 | 0.52 |
| Upper 95% CI Coefficient | 1.58 | 2.47 | 0.87 | 1.15 | 2.02 | 1.22 | 0.79 | 1.69 | 0.02 | 0.16 | 0.07 | 0.97 | 1.48 |
|  |  |  |  |  |  |  |  |  |  |  |  |  |  |
| **Gait disturbance** |  |  |  |  |  |  |  |  |  |  |  |  |  |
| Coefficient | 3.13 | 0.45 | 0.73 | 1.75 | -1.05 | 0.81 | 0.11 | 0.00 | 0.00 | 0.20 | -0.12 | 0.59 | 0.10 |
| Standard Error | 0.85 | 0.79 | 0.53 | 0.77 | 0.77 | 0.60 | 0.62 | 0.97 | 0.01 | 0.25 | 0.02 | 0.29 | 0.34 |
| Z | 3.70 | 0.57 | 1.38 | 2.29 | -1.36 | 1.34 | 0.18 | 0.00 | 0.22 | 0.81 | -5.28 | 2.04 | 0.31 |
| P | 0.00 | 0.57 | 0.17 | 0.02 | 0.18 | 0.18 | 0.86 | 1.00 | 0.83 | 0.42 | 0.00 | 0.04 | 0.76 |
| Lower 95% CI Coefficient | 1.47 | -1.10 | -0.30 | 0.25 | -2.57 | -0.37 | -1.10 | -1.90 | -0.02 | -0.29 | -0.17 | 0.02 | -0.56 |
| Upper 95% CI Coefficient | 4.79 | 1.99 | 1.76 | 3.25 | 0.47 | 1.99 | 1.32 | 1.90 | 0.03 | 0.69 | -0.08 | 1.16 | 0.77 |
|  |  |  |  |  |  |  |  |  |  |  |  |  |  |
| **Limb tone** |  |  |  |  |  |  |  |  |  |  |  |  |  |
| Coefficient | 1.01 | 0.04 | 0.35 | 0.76 | -0.07 | -0.02 | 0.06 | -0.72 | 0.00 | -0.32 | -0.12 | -0.09 | 0.00 |
| Standard Error | 0.77 | 0.55 | 0.39 | 0.59 | 0.70 | 0.46 | 0.45 | 0.67 | 0.01 | 0.18 | 0.02 | 0.21 | 0.24 |
| Z | 1.31 | 0.08 | 0.89 | 1.29 | -0.09 | -0.05 | 0.14 | -1.08 | 0.20 | -1.76 | -7.54 | -0.42 | 0.00 |
| P | 0.19 | 0.94 | 0.37 | 0.20 | 0.93 | 0.96 | 0.89 | 0.28 | 0.84 | 0.08 | 0.00 | 0.67 | 1.00 |
| Lower 95% CI Coefficient | -0.50 | -1.04 | -0.42 | -0.39 | -1.44 | -0.92 | -0.82 | -2.03 | -0.02 | -0.67 | -0.15 | -0.50 | -0.46 |
| Upper 95% CI Coefficient | 2.53 | 1.12 | 1.12 | 1.91 | 1.31 | 0.87 | 0.95 | 0.59 | 0.02 | 0.04 | -0.09 | 0.32 | 0.46 |
|  |  |  |  |  |  |  |  |  |  |  |  |  |  |
| **Limb coordination** |  |  |  |  |  |  |  |  |  |  |  |  |  |
| Coefficient | 0.86 | 0.07 | -0.60 | -0.02 | 0.76 | -0.59 | -0.09 | 1.29 | 0.02 | 0.10 | -0.31 | -0.80 | -1.26 |
| Standard Error | 0.81 | 0.59 | 0.40 | 0.60 | 0.75 | 0.54 | 0.46 | 0.78 | 0.01 | 0.20 | 0.02 | 0.24 | 0.27 |
| Z | 1.06 | 0.12 | -1.49 | -0.04 | 1.02 | -1.10 | -0.20 | 1.65 | 2.03 | 0.51 | -13.46 | -3.38 | -4.65 |
| P | 0.29 | 0.90 | 0.14 | 0.97 | 0.31 | 0.27 | 0.84 | 0.10 | 0.04 | 0.61 | 0.00 | 0.00 | 0.00 |
| Lower 95% CI Coefficient | -0.72 | -1.09 | -1.38 | -1.19 | -0.71 | -1.64 | -1.01 | -0.24 | 0.00 | -0.30 | -0.36 | -1.26 | -1.79 |
| Upper 95% CI Coefficient | 2.43 | 1.23 | 0.19 | 1.14 | 2.24 | 0.46 | 0.82 | 2.83 | 0.04 | 0.50 | -0.27 | -0.33 | -0.73 |
|  |  |  |  |  |  |  |  |  |  |  |  |  |  |
| **Supranuclear gaze palsy** |  |  |  |  |  |  |  |  |  |  |  |  |  |
| Coefficient | 1.03 | -0.16 | 0.37 | 1.74 | -0.90 | 0.17 | 0.32 | -0.03 | 0.01 | -0.11 | -0.19 | -0.46 | -0.12 |
| Standard Error | 0.74 | 0.61 | 0.39 | 0.60 | 1.14 | 0.48 | 0.52 | 0.61 | 0.01 | 0.18 | 0.02 | 0.21 | 0.22 |
| Z | 1.41 | -0.27 | 0.95 | 2.91 | -0.79 | 0.35 | 0.61 | -0.05 | 0.77 | -0.61 | -11.15 | -2.15 | -0.51 |
| P | 0.16 | 0.79 | 0.34 | 0.00 | 0.43 | 0.72 | 0.54 | 0.96 | 0.44 | 0.54 | 0.00 | 0.03 | 0.61 |
| Lower 95% CI Coefficient | -0.41 | -1.35 | -0.39 | 0.57 | -3.14 | -0.76 | -0.70 | -1.23 | -0.01 | -0.46 | -0.22 | -0.87 | -0.56 |
| Upper 95% CI Coefficient | 2.47 | 1.03 | 1.14 | 2.91 | 1.34 | 1.10 | 1.33 | 1.17 | 0.03 | 0.24 | -0.16 | -0.04 | 0.33 |
|  |  |  |  |  |  |  |  |  |  |  |  |  |  |
| **Nystagmus** |  |  |  |  |  |  |  |  |  |  |  |  |  |
| Coefficient | -0.17 | 0.92 | -0.05 | 0.05 | 0.47 | 0.11 | 0.07 | -0.08 | 0.02 | -0.03 | -0.12 | -0.66 | -0.37 |
| Standard Error | 0.89 | 0.56 | 0.42 | 0.70 | 0.84 | 0.48 | 0.53 | 0.65 | 0.01 | 0.19 | 0.02 | 0.23 | 0.24 |
| Z | -0.19 | 1.64 | -0.11 | 0.07 | 0.56 | 0.23 | 0.12 | -0.12 | 1.90 | -0.16 | -7.09 | -2.88 | -1.55 |
| P | 0.85 | 0.10 | 0.91 | 0.94 | 0.57 | 0.82 | 0.90 | 0.91 | 0.06 | 0.87 | 0.00 | 0.00 | 0.12 |
| Lower 95% CI Coefficient | -1.92 | -0.18 | -0.87 | -1.31 | -1.17 | -0.84 | -0.98 | -1.36 | 0.00 | -0.39 | -0.15 | -1.11 | -0.83 |
| Upper 95% CI Coefficient | 1.58 | 2.01 | 0.77 | 1.41 | 2.11 | 1.06 | 1.11 | 1.21 | 0.04 | 0.33 | -0.09 | -0.21 | 0.10 |
|  |  |  |  |  |  |  |  |  |  |  |  |  |  |
| **Alien limb phenomenon** |  |  |  |  |  |  |  |  |  |  |  |  |  |
| Coefficient | -0.85 | -1.15 | -2.10 | -0.21 | -13.15 | -0.05 | 0.39 | 0.16 | 0.00 | -0.04 | -0.05 | -0.85 | -1.07 |
| Standard Error | 1.16 | 1.08 | 1.04 | 0.78 | 606.37 | 0.54 | 0.53 | 0.83 | 0.01 | 0.23 | 0.02 | 0.29 | 0.34 |
| Z | -0.73 | -1.06 | -2.02 | -0.27 | -0.02 | -0.09 | 0.72 | 0.19 | 0.08 | -0.18 | -2.64 | -2.89 | -3.13 |
| P | 0.46 | 0.29 | 0.04 | 0.79 | 0.98 | 0.93 | 0.47 | 0.85 | 0.93 | 0.86 | 0.01 | 0.00 | 0.00 |
| Lower 95% CI Coefficient | -3.13 | -3.27 | -4.13 | -1.74 | -1201.60 | -1.11 | -0.66 | -1.46 | -0.02 | -0.48 | -0.09 | -1.42 | -1.74 |
| Upper 95% CI Coefficient | 1.42 | 0.97 | -0.07 | 1.31 | 1175.31 | 1.01 | 1.43 | 1.77 | 0.03 | 0.40 | -0.01 | -0.27 | -0.40 |
