# Supplementary material for: Prevalence and Treatments of Movement Disorders in Prion Diseases: A Longitudinal Cohort Study
Source: Mov Disord. 2022 Jul 16;37(9):1893–903. doi: 10.1002/mds.29152 (PMC9543300; doi:10.1002/mds.29152)
Supplement: Supplementary file 5 — Table S2 Results of ordinal logistic regression model for investigations. We fitted models that included age, Medical Research Council Prion Disease Rating Scale (MRC Scale), gender, disease type, and investigation status to predict each movement disorder (recorded as absent/present/unassessable). Each investigation was recorded as abnormal/normal/not assessed. Coefficients for the effect of the investigation are shown in each cell (in orange, P = 0.01–0.05; in yellow, P = 0.001–0.01; no colour, P > 0.05). Taking multiple testing into account we concluded no statistically significant associations (P < 0.001). [file MDS-37-1893-s004.docx]

| Investigation | n | pursuit | saccades | bradykinesia | myoclonus | chorea | tremor | gait | tone | coordination | supranuclear ophthalmoparesis | nystagmus | alien limb |
| --- | --- | --- | --- | --- | --- | --- | --- | --- | --- | --- | --- | --- | --- |
| Cortical Ribbon | 650 | -0.20 | 0.22 | 0.08 | 0.09 | -0.76 | -0.19 | -0.94 | -0.35 | -0.15 | -0.23 | 0.14 | 0.16 |
| Pulvinar sign | 625 | -0.30 | -0.27 | 0.33 | -0.90 | 0.46 | -1.44 | 1.60 | -1.06 | 0.33 | 0.40 | 0.44 | 0.82 |
| Basal Ganglia | 660 | -0.24 | -0.35 | -0.26 | 0.26 | -0.28 | -0.13 | -0.46 | -0.19 | 0.07 | -0.53 | -0.11 | -0.28 |
| Thalamus | 539 | -0.27 | -0.44 | -0.47 | 0.35 | -0.09 | -0.11 | -0.41 | -0.07 | -0.42 | -0.43 | -0.35 | -0.04 |
| PSWCs | 471 | 0.27 | 0.26 | 0.06 | -0.15 | 0.61 | -0.04 | -0.13 | -0.43 | 0.50 | 0.24 | 0.84 | -0.1 |
| Generalised slowing | 279 | 0.56 | 0.67 | 0.32 | 0.46 | -0.36 | -0.37 | -0.65 | 0.15 | 0.13 | 0.81 | 0.26 | -0.57 |
| 14--3--3 | 391 | -0.39 | -0.29 | 0.02 | 0.58 | -0.02 | 0.25 | 0.54 | 0.54 | 0.75 | -0.27 | 0.30 | -0.29 |
| Rt-QuIC | 115 | -0.11 | -1.69 | -1.84 | 0.42 | n/a | 1.33 | 1.96 | 0.23 | 2.57 | 0.20 | 0.33 | -0.23 |

**Table S2. Results of ordinal logistic regression model for investigations.** We fitted models that included age, MRC Scale, gender, disease type, and investigation status to predict each movement disorder (recorded as absent/present/unassessable). Each investigation was recorded as abnormal/normal/not assessed. Coefficients for the effect of the investigation are shown in each cell (in orange, P=0.01-0.05; in yellow, P=0.001-0.01; no colour, P>0.05). Taking multiple testing into account we concluded no statistically significant associations (P<0.001).
